# The so-called ligamentum arteriosum is innervated

**DOI:** 10.1371/journal.pone.0291879

**Published:** 2023-09-21

**Authors:** Quaye Benedicta

**Affiliations:** 1 Department of Clinical Anatomy, Faculty of Health and Medicine, Lancaster University, Lancaster, United Kingdom; 2 Department of Cardiopulmonary Neurobiology, Institute of Anatomy and Cell Biology, Justus Liebig University, Giessen, Germany; Keio University - Shinanomachi Campus: Keio Gijuku Daigaku - Shinanomachi Campus, JAPAN

## Abstract

The ductus arteriosus is a muscular artery connecting the pulmonary trunk directly to the aorta in fetal circulation in order to by-pass the fluid filled lungs. Post-natally, this vessel is speculated to undergo obliteration, fibrosis and ultimately metamorphosize into a band of ligament, thereby changing name from the ductus arteriosus to the ligamentum arteriosum (LA). Earlier studies into the innervation of the ductus arteriosus reported innervation from the left aortic and vagus nerves. However, information of what becomes of the innervation is scanty and contradictory. I hypothesized that; this fetal shunt still receives innervation even in post-uterine life. To test this, LA of human, pig, and wild-type mice were studied using double-immunofluorescence labeling using antibodies directed against structural and general neuronal marker proteins (Smooth muscle actin and Protein gene product 9.5 (PGP 9.5, respectively). Additionally, TEM studies were performed on mouse LA. Results from the present study demonstrates an extensive innervation of the LA in animals (mice and pigs) and in senescent humans validated by two independent methods, i.e., immunolabeling with antibody directed against PGP 9.5 and TEM. Intense immunoreactivity was clearly visible in samples subjected to PGP-immunolabeling. TEM revealed the presence of nerve terminals with about 30% of all nerve terminals observed less than 1 μm away from smooth muscle cells within the LA. This clearly differs from elastic arteries, where the distance between autonomic terminals and smooth muscle cells is rarely less than 1 μm. Conceivably, these results imply that the so- called LA receives innervation representative of that present within the ductus arteriosus during fetal life. This provides the first reliable study of innervation of the LA and makes room for further investigation into the neurochemistry of this innervation. This is crucial as the presence of nerve terminals may play a role in vessel compliance or impedance of the two great vessels related to this structure. The substances released by these fibers may also have an influence on cells and tissues in the immediate microenvironment of this structure.

## Introduction

**Fig 1:**
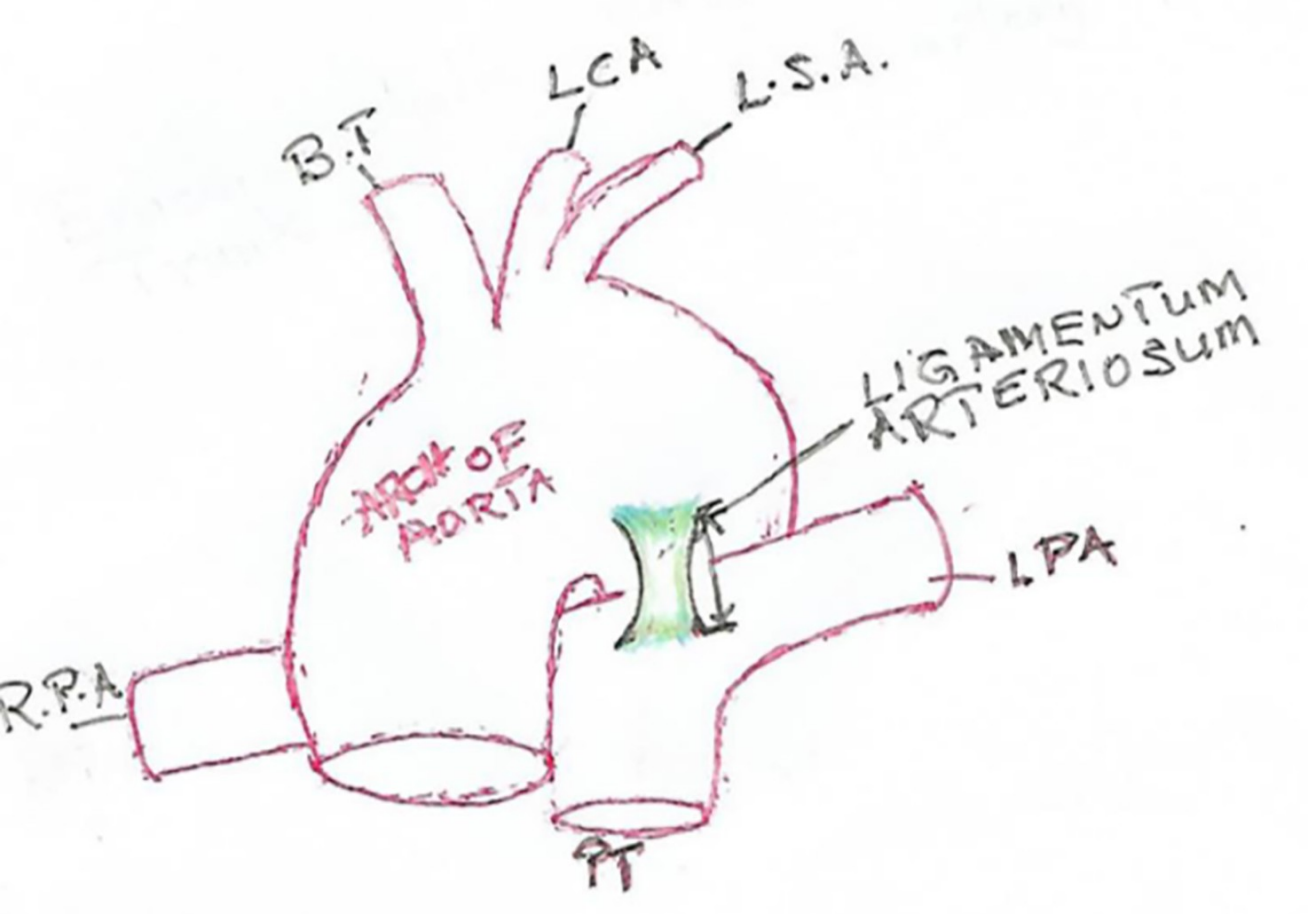
LA and its attachment to the pulmonary trunk (PT) and the aortic arch. The PT giving off the left pulmonary artery (LPA) and right pulmonary artery (RPA). The aortic arch also giving off the first 3 branches brachiocephalic trunk (BT), left carotid (LCA) and left subclavian artery (LSA). Green, blue and yellow colour within LA indicates areas of intense innervation in the structure.

The ductus arteriosus is the muscular artery connecting the pulmonary trunk directly to the aorta in fetal circulation to by-pass the fluid filled lungs and derived from the sixth branchial arch arteries of the embryo [1]. Post-natally, this vessel is speculated to undergo obliteration, fibrosis, and metamorphosis to a band of ligament, thereby changing name to the ligamentum arteriosum (Fig 1). A recent re-investigation into the morphology of the LA reported the presence of contractile muscular elements present within this structure [2]. Though the LA has long been described as a band of fibrotic tissue, the point of attachment or insertion of the structure to the great vessels have been implicated in the pathophysiology of aortic coarctation, the congenital birth defect [3, 4].

Earlier histological reports of nerve fibers within the wall of the ductus arteriosus have been described by various investigators [5–7]. Boyd in 1941 conducted studies into the innervation of the ductus arteriosus and reported innervation from the left aortic and vagus nerves. He speculated that the vagus contributed chiefly to the innervation of the antero-inferior aspect of the vessel while the aortic nerve was concerned mainly with the innervation of its postero-superior surface, though in a few specimens, the inferior aspect of the ductus arteriosus received some fibers from the recurrent laryngeal nerve as this nerve hooks under it [6]. Presumably, these fibers were vagal fibers leaving the nerve at a lower level than is usual and which accompany the recurrent nerve for a short distance, he added. In the younger embryos, however, the fibers tend to penetrate further into the wall of the ductus, approaching, and sometimes reaching, the intima. The fibers which reach the intima usually bend back into the muscular coat of the ductus. In the older embryos, the fibers are for the most part restricted to the outer third of the media and to the adventitia, but a few fibers can always be traced more deeply into the wall of the ductus. Another study reports the persistence of a few of the nerve endings within the ductus arteriosus [8]. A rare report did not explicitly state that he found such endings in the LA of the adult rabbit but his statements suggest that he did for he writes *’the possible function of nerve-endings in the wall of the obliterated duct (LA of the adult) can only be surmised’* [9].

Conclusive experiments or reports about what happens to the innervation of the ductus arteriosus at birth when the circulation of blood through it ceases are scanty. This study aims to fill the blank in knowledge and literature regarding this. Based on this premise, I ask, in post-uterine life, does this fetal shunt still receives innervation?

## Materials and methods

### Samples used

#### Mouse

Wild type mice (C57BL/6J, n = 8 aged between 8 to 30 weeks) from Janvier laboratories (Le Genest-Saint-Isle, France) were housed under specific pathogen free (SPF) conditions (10 h dark, 14 h light) with free access to food and water. This study was carried out in accordance with the recommendations of the European Communities Council Directive of 24 November 1986 (86/609/EEC). The protocol was approved by the local authorities, i.e., Regierungspräsidium Giessen, Germany (reference no. 714_M). All samples were taken after mice were killed by inhalation of an overdose of 5% isoflurane (Abbott, Wiesbaden, Germany) and exsanguination through abdominal blood vessels.

#### Pig

Mediastinal blocks of pigs were acquired from a commercial abattoir. The abattoir had a qualified veterinary doctor who thoroughly examined the pigs after the slaughter. The pigs were only females (n = 10) aged between 6 months to one year. The mediastinal blocks were immediately kept on ice and transported to the laboratory in an ice cooler. The LA was dissected and excised from its attachments to the aorta and pulmonary trunk.

#### Human

Human LA from body donors of the anatomy dissection course in the Justus Liebig University was dissected and excised with its attachment to the two major vascular segments to which it is attached. There were six females and four males aged between 67–97 years. Registration as a body donor in Germany is only possible from the age of sixty. The ligaments were immersed in Zamboni fixative for one day to refix them even though they had already been fixed for the dissection course with a fixative (comprising of 10 liters (l) of 90% ethanol (57%), 1.3 liters (l) of 37% formalin (3%), 0.8 l of phenoxyethanol (5%) and 0.8 l of glycerin (5%)). These were all added to 3 liters (l) of water. The embalming process of donors takes between two to four hours after its arrival at the institute. After embalming, the donors usually remain in the Anatomy for two to three years, unless in the case of registration as a permanent donor, which stays in the long term. None of the cadavers used were permanent donors and had been embalmed for 3 months prior to the start of the dissection course.

Cryoprotection (Pig and mouse samples only) and sample processing for immunofluorescence staining.

In mouse, harvested mediastinal blocks containing the LA, and in pig, the LA dissected and excised from its attachment to the aorta and pulmonary trunk, were placed in Zamboni fixative for about 5–6 h. The solution was then replaced by 0.1 M phosphate buffer and changed every hour until fluid around the sample was clear. The sample was then incubated overnight in 18% sucrose in 0.1 M phosphate buffer and kept at 4°C. The samples were cryofixed using optimum cutting temperature (OCT) compound (tissue tek, Sakura Finetek, Staufen, Germany) and 2-methylbutane solution (Carl Roth GmbH, Germany) which had been chilled using liquid nitrogen. Samples subjected to processing protocol described above were used for negative and positive control for all immunofluorescent staining.

Frozen mouse and pig sections were allowed to air-dry for 1 h at room temperature. Then they were covered for 1 h with blocking medium (Table 1) and subsequently incubated with primary antibody (Table 2). The primary antibody was dissolved in phosphate buffered saline (PBS) with addition of NaN3+S (0.01% NaN3 and 0.05 M NaCl) in the working dilution. Double-immunolabelling was performed by applying primary antibody against αSMA conjugated to fluorescein isothiocyanate (FITC) overnight at room temperature, followed by washing steps (2 × 10 min in PBS) and subsequent 1 h incubation with secondary antibody conjugated to cyanine 3 (Cy3, Table 3) targeted at primary antibodies against antigens other than αSMA. This was followed by PBS washes (2 × 10 min). The sections were fixed in 4% paraformaldehyde (PFA) for 10 min, followed by a final washing in PBS (2 x 10 min). Finally, sections were covered in carbonate-buffered glycerol (Carl Roth GmbH and Co, Karlsruhe, Germany) at pH 8.6. Evaluation and measurements were done using an epifluorescence microscope (BX 60, Olympus, Hamburg, Germany or Axioplan 2 imaging, Zeiss, Jena, Germany) equipped with cameras (Olympus DP73 and AxioCam MRm, Germany) connected to Cell Sens Dimension 2.1 and AxioVision Rel 22 4.8.2 SPZ software’s respectively. The microscope was equipped with the appropriate filter combinations (Table 4).

**Table 1:** Buffers and solutions used.

Zamboni solution	2% paraformaldehyde, 15% saturated picric acid in 0.1 M phosphate buffer, pH 7.4
0.1 M phosphate buffer	0.1 M NaH2PO4 x 2H2O 31.2 g/l, 0.1 M Na2HPO4 x 2H2O 35.6 g/l, pH 7.4
18% sucrose	99.5% saccharose + 0.1 M Phosphate buffer
blocking solution	10% normal pork serum, 0.1% bovine serum albumin (BSA), 0.5% Tween 20 in PBS
buffered glycerol	solution A: 1.5 M Na2CO3, solution B: 1.5 M Na2CO3 pH 8.6, solution C: 100% glycerol
phosphate buffered salt	solution (PBS) solution A: NaH2PO4 x 2H2O 28.75 ml, solution B: Na2HPO4 x 2H2O 96.2 ml, pH 7.4, 22.4 g NaCl
PBS+S	solution A: NaH2PO4 x 2H2O 28.75 ml, solution B: Na2HPO4 x 2H2O 96.2 ml, pH 7.4, 44.8 g NaCl
4% paraformaldehyde	paraformaldehyde, 0.1 M phosphate buffer
citric acid	9.62 g/l citric acid, 13 pellets NaOH, 2 M NaOH, pH 6.0

**Table 2:** Primary antibodies used. P = polyclonal, M = monoclonal.

Antigen	Host species	Dilution	Source	Code
Protein gene product 9.5 (PGP)	Rabbit (P)	1:8000	BioTrend, Germany	040112
Alpha smooth muscle actin (αSMA)	Mouse (M)	1:1000	Sigma-Aldrich Germany	F3777

**Table 3:** Secondary antibodies used.

Antigen	Conjugate	Host species	Dilution	Catalogue number	Source
Rabbit Ig	Cy3	Donkey	1:2000	AP182C	Merck, Germany
Rabbit Ig	Alexa 488	Donkey	1:500	A21206	Thermo Fisher, Germany

**Table 4:** Fluorochrome filters and excitation wavelengths in fluorescence microscopy.

Fluorochrome	Colour	Excitation filter (nm)	Barrier filter (nm)
FITC/ Alexa 488	green	460–490	515–550

Paraffin embedded human LA sections were deparaffinized using xylene (VWR Chemicals, France), then rehydrated by passing it through the graded alcohol in decreasing order, i.e., absolute alcohol, 96% ethanol, then 70% ethanol (all from the same manufacturer, Sigma-Aldrich, Germany). Slides were then washed with PBS and put in a cuvette containing 10 μl of citric acid buffer. The cuvette is then put in a bowl containing 700mls of aqua and brought to a boil in a standard microwave at 700w for 15 to 20mins. It is then left to cool down, sections are removed from citric acid and incubated in PBS for 5mins. Sections are dried, covered for 1 h with blocking medium (10% normal pork serum, 0.1% bovine serum albumin (BSA), 0.5% Tween 20 in PBS) and followed through with immunostaining procedure for frozen sample as described prior.

### Transmission electron microscopy (Mouse only)

Mice and pig LA (n = 8 for both) were fixed for at least 24 h in 2% paraformaldehyde and 1.5% glutaraldehyde (Merck) in 0.1 M phosphate buffer (pH 7.4). After fixation, specimens were washed in HEPES buffer 0.15 M, pH 7.4 (5 × 10 min), osmicated for 2 h in aqueous 1% osmium tetroxide (Sigma-Aldrich), washed in distilled water, contrasted in 1% uranyl acetate (Merck) overnight, and embedded in epon (Agar Scientific, Essex, UK). Ultrathin sections (between 70–90 nm) were cut using an ultramicrotome (Reichert Ultracut E, Leica). The ultrathin sections were viewed using a transmission electron microscope (EM 902 N, Zeiss, Germany) equipped with a slow scan 2 K CCD camera (TRS, Tröndle, Moorenweis, Germany) connected to Image SP software version 1.2.8.57 (Unitary enterprise “SYSPROG”).

### Ethics approval statement

Animal welfare: This study was carried out in accordance with the recommendations of the European Communities Council Directive of 24 November 1986 (86/609/EEC). The protocol was approved by the local authorities, i.e., Regierungspräsidium Giessen, Germany (reference no. 714_M). The body donors sign an official testamentary disposition protected by the Art. 6 Para. 1 a of the European General Data Protection Regulation to leave their body to the anatomy institute after death for the training of young doctors, the further and further training of (specialist) doctors and science (https://www.unigiessen.de/fbz/fb11/institute/anatomie/koerperspende). A formal written approval from the university was also obtained prior to acquisition of pig samples from the commercial abattoir which had an in house vertinary doctor to ensure the right protocol was followed.

### Statistical analysis

Nerve fibers with clearly visible vesicles were included for the morphoquantitative analysis to study distance from nerve terminals to nearest myocyte. Smooth muscle cells also had the characteristic needed to fit the criteria for smooth muscle classification. The distance was measured using software embedded within the Image SP software version 1.2.8.57. Figures obtained were imputed into GraphPad Prism and histograms generated from it. Histogram reported the percentage representation of the number of nerve terminals with respect to their distance from the nearest myocyte in transmission electron microscopy of murine.

## Results

### Double-labelling immunofluorescence using antibodies against structural and general neuronal marker proteins in mouse

**Fig 2:**
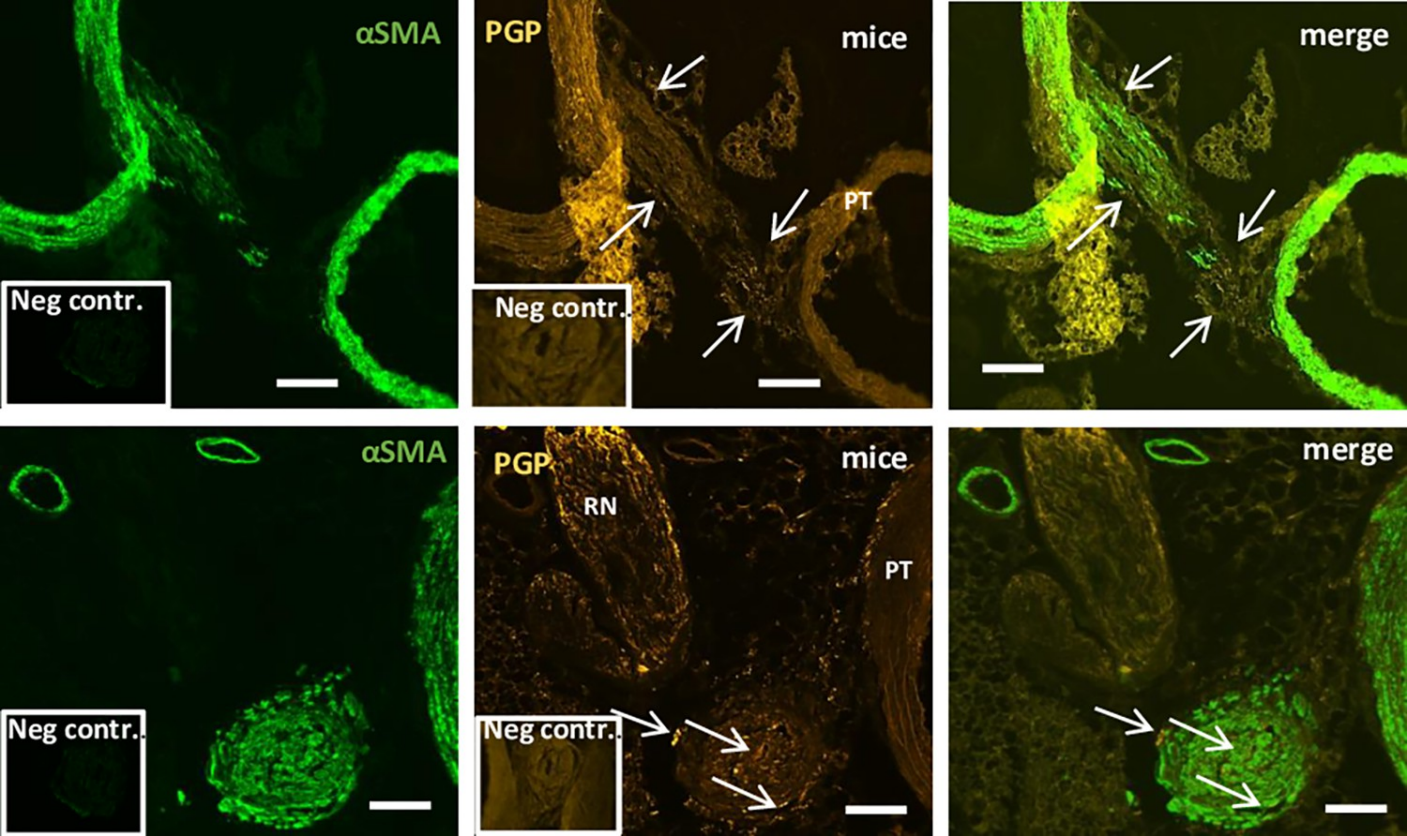
Double-immunolabeling of murine LA in longitudinal and cross sections. In upper panel, first picture reveals the LA attached to the aorta and pulmonary trunk (PT) with PGP-positive fibres along the external borders, at the point of attachments to the pulmonary trunk (white arrows). Middle picture shows immunoreactivity against αSMA. The last picture is a merge showing PGP-positive fibres close to smooth muscle cells. Controls run without primary antibody (neg. contr.) shown in the inserts. In lower panel, first picture shows PGP-positive fibres (white arrows) present around the periphery, within the core and also at the point of attachment to the pulmonary trunk (PT). Middle picture shows presence of immunoreactivity to αSMA. Last picture is a merge of the first and middle picture. Control run without primary antibody directed against PGP9.5 (neg. contr.) shown in the insert. Scale bar = 50 μm.

Immunohistochemistry was used to assess the general innervation of the LA. Cross-sectionally and longitudinally sectioned LA sections were subjected to double-immunolabeling directed against PGP9.5 and FITC-conjugated monoclonal antibodies directed against αSMA. PGP9.5-positive fibers were observed around the periphery, within the musculature and at the point of attachment to the major vessels to which the LA was attached (Fig 2). To determine the specificity of the secondary antibody, primary antibodies were omitted and replaced by PBS There was no reactivity in the LA when primary antibody was omitted.

### Double-labelling immunofluorescence using antibodies against structural and general neuronal marker proteins in pig and human

**Fig 3:**
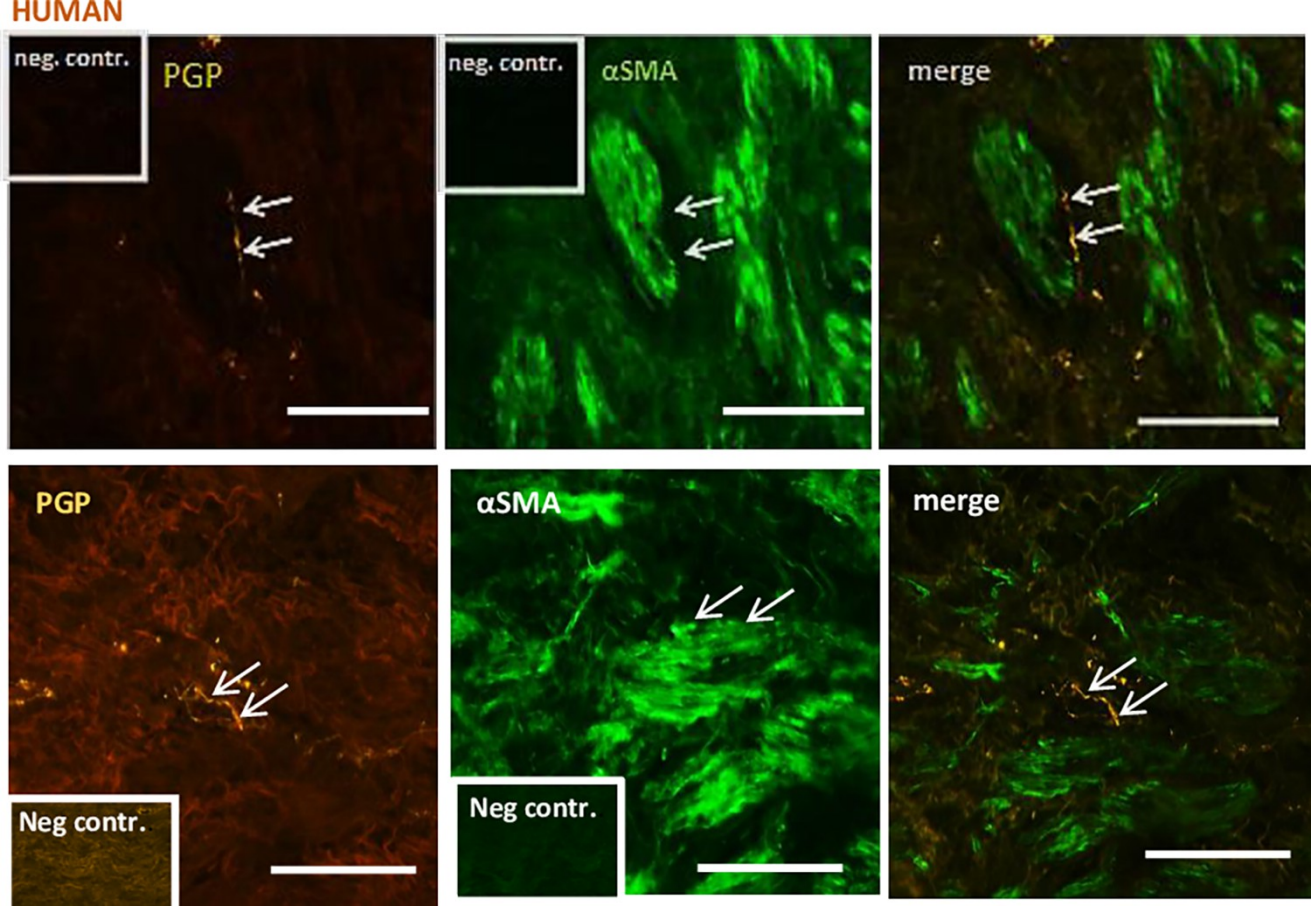
Double-immunolabeling of human LA. In upper panel, first picture on the left showing numerous PGP-immunoreactive fibres (white arrows) present within the LA. Middle picture shows immunoreactivity against αSMA. The last picture is a merge showing PGP-positive fibres close to smooth muscle cells. Controls run without primary antibody (neg. contr.) shown in inserts. Scale bar = 50 μm.

**Fig 4:**
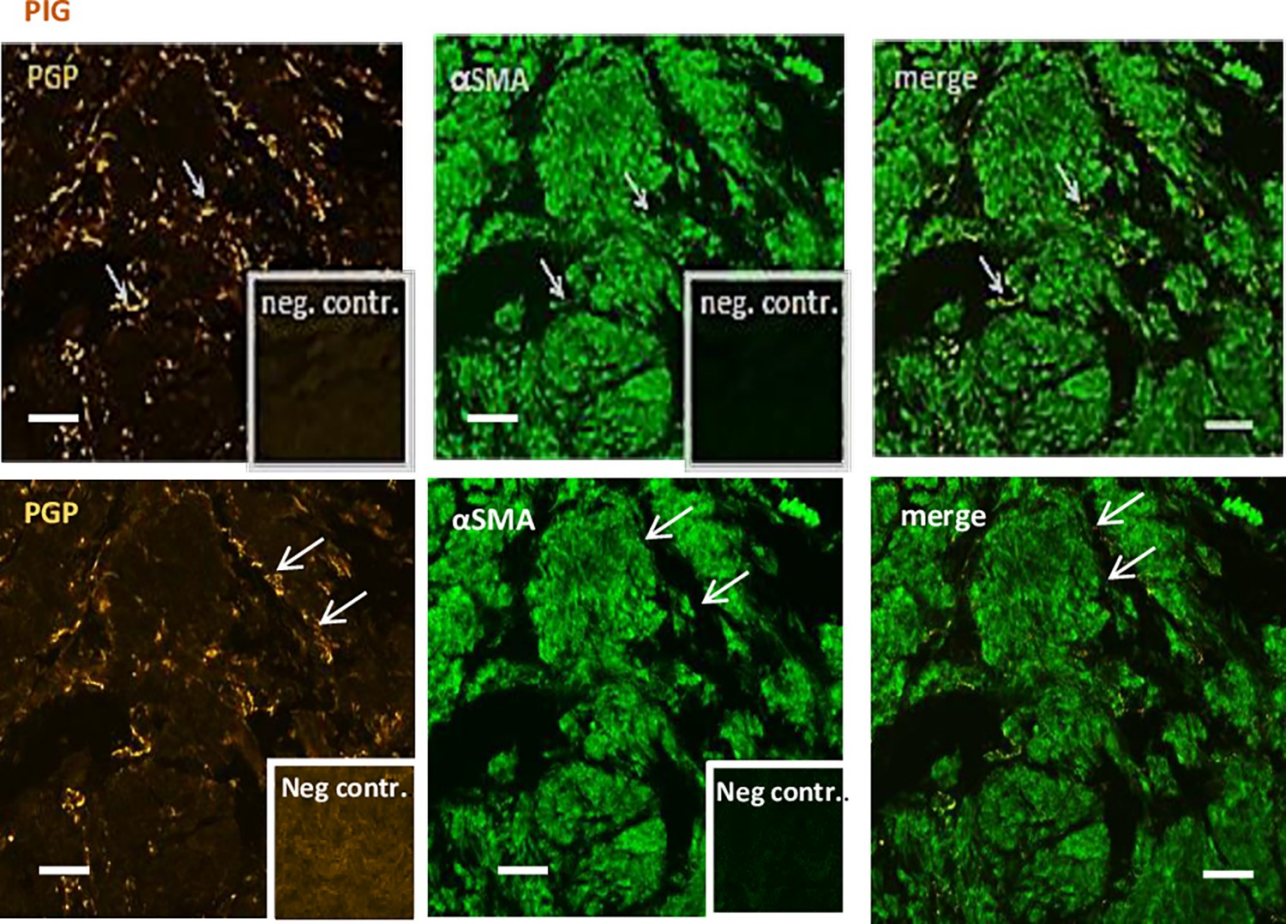
Double-immunolabeling of pig. In panel, first picture shows PGP-immunoreactive nerve fibres (white arrows). The middle picture shows immunoreactivity against αSMA. The last picture is a merge. Controls run without primary antibodies (neg. contr.) shown in the inserts. Scale bar = 50 μm.

Immunohistochemistry used to assess the general innervation of the LA in pig and human using antibodies against PGP reported immunoreactive nerve fibers. PGP-positive fibers were detected along bundles of smooth muscle cells and within the musculature of the LA (Figs 3 and 4). To determine the specificity of the secondary antibody, primary antibodies were omitted and replaced by PBS. There was no reactivity in the LA when primary antibody was omitted (Figures inserts).

To assess the general innervation of LA in human, double-labelling immunohistochemistry was performed using primary antibodies directed against PGP and a FITC-conjugated monoclonal antibody against αSMA. PGP-Positive fibers were detected along bundles of smooth muscle cells and within the musculature of the LA (Fig 3). To determine the specificity of the secondary antibody, primary antibodies were omitted and replaced by PBS (Fig 2 inserts). There was no reactivity in the LA when primary antibody was omitted (Fig 2 inserts). Similar reactivity was reported in pig samples (Fig 4) subjected to the same staining.

### TEM of smooth muscle innervation in mice

**Fig 5:**
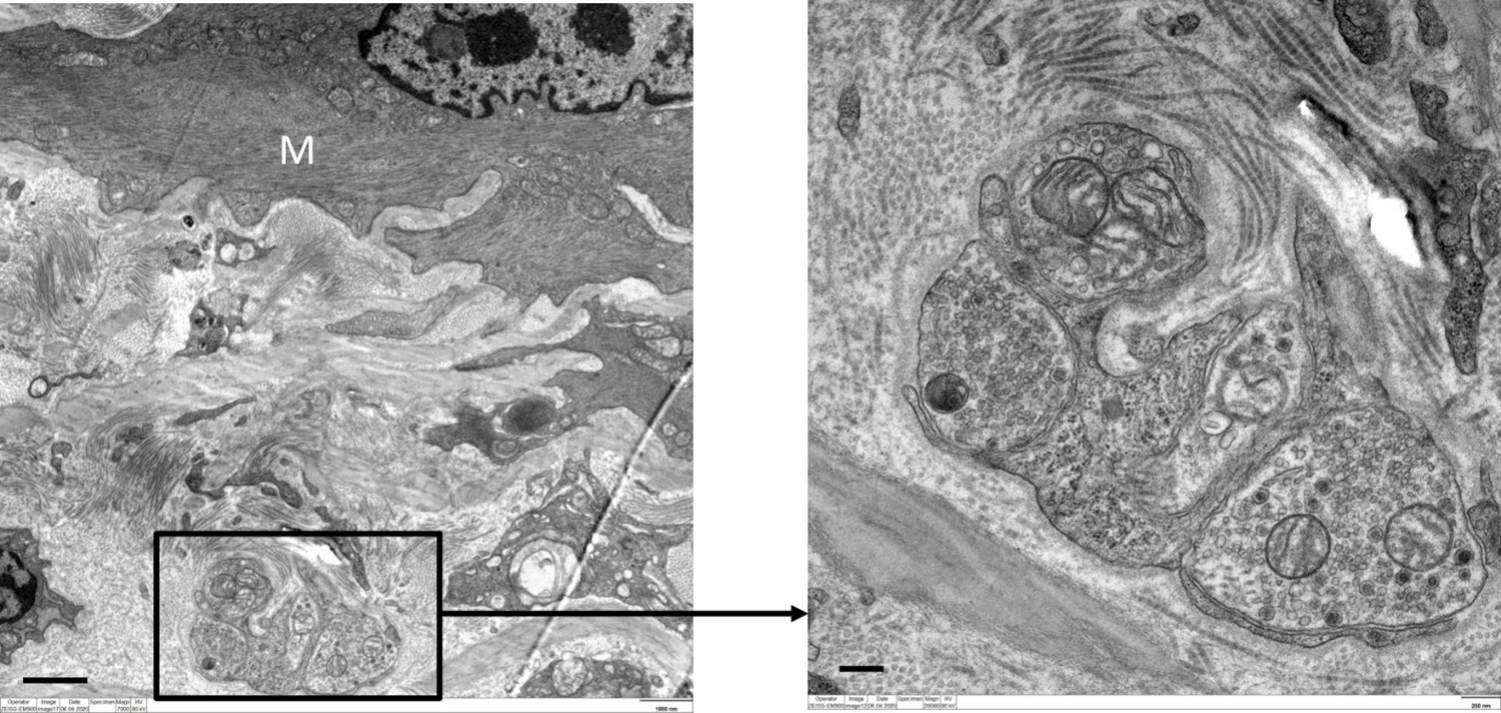
TEM of murine LA revealing nerve fiber bundle (boxed area) ˃2 μm away from a myocyte (M). Scale bar = 1 μm.

**Fig 6:**
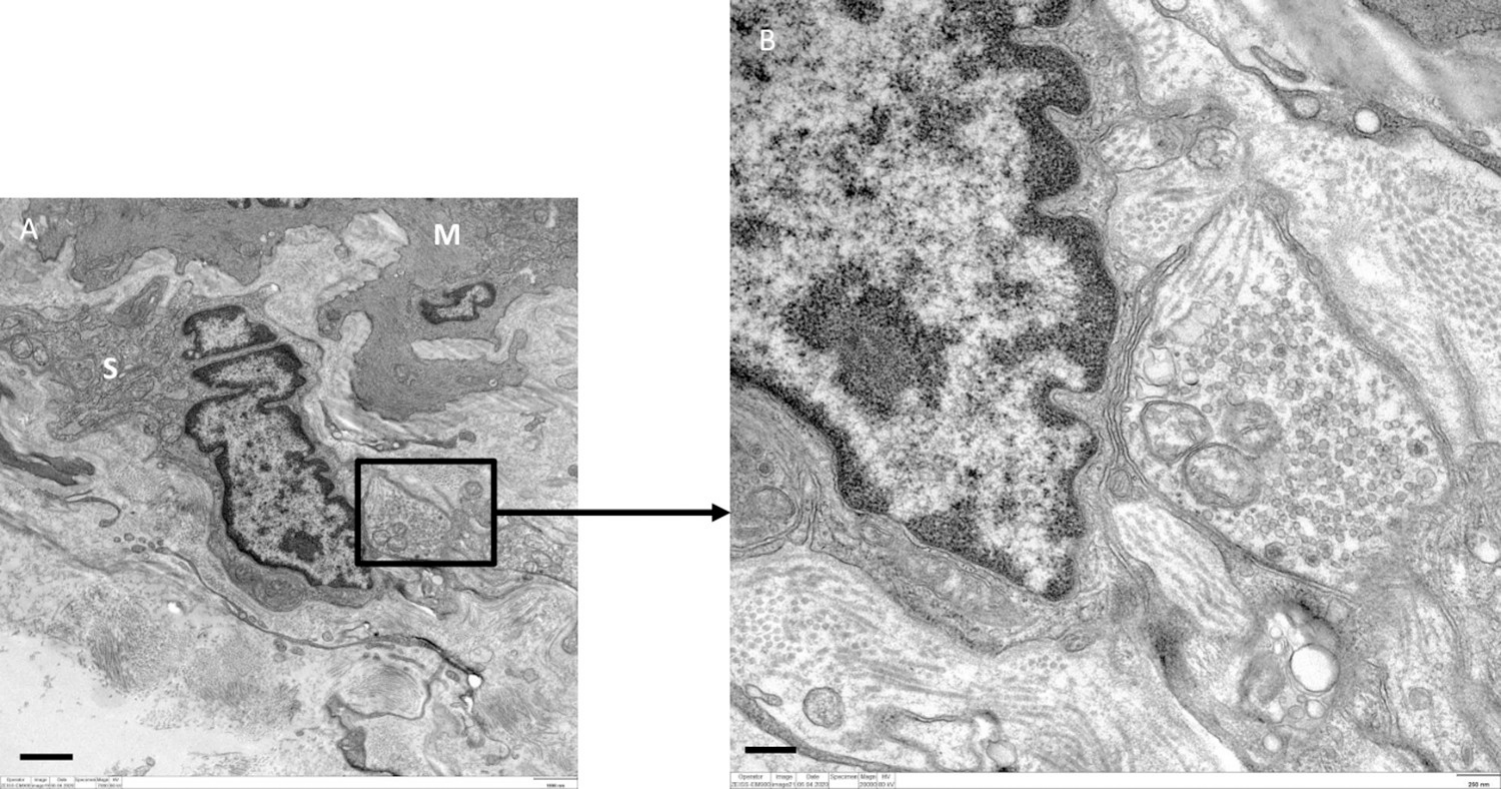
TEM of murine LA with a nerve terminal (boxed area) encapsulated in its Schwann cell (S) ˃ 0.8 μm away from a myocyte (M). Scale bars: A = 0.5 μm.

**Fig 7:**
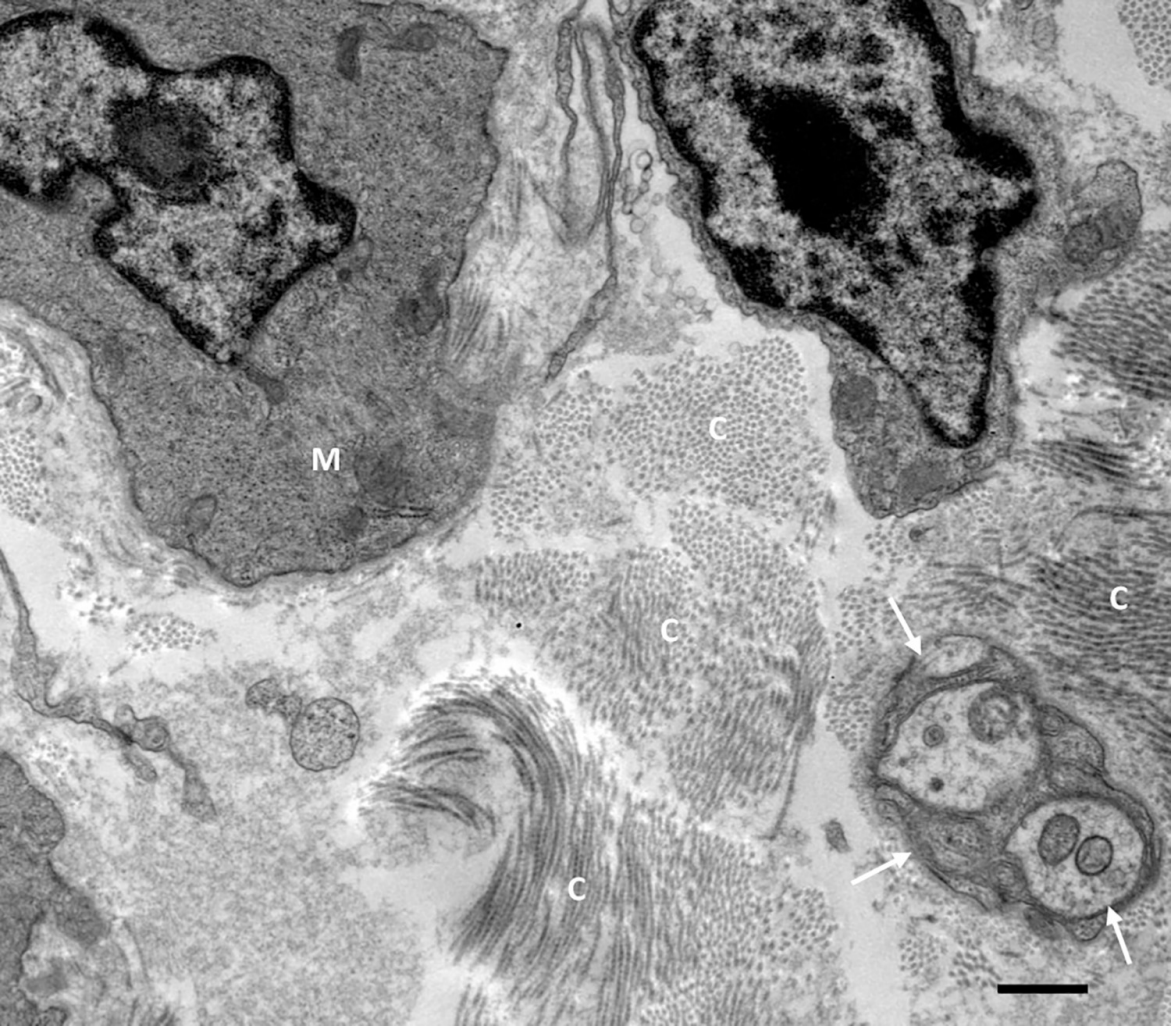
TEM of murine LA with collagen fibrils (C) found dispersed between a nerve fiber bundle (white arrows) and a myocyte (M). Scale bars: A = 0.5 μm.

**Fig 8:**
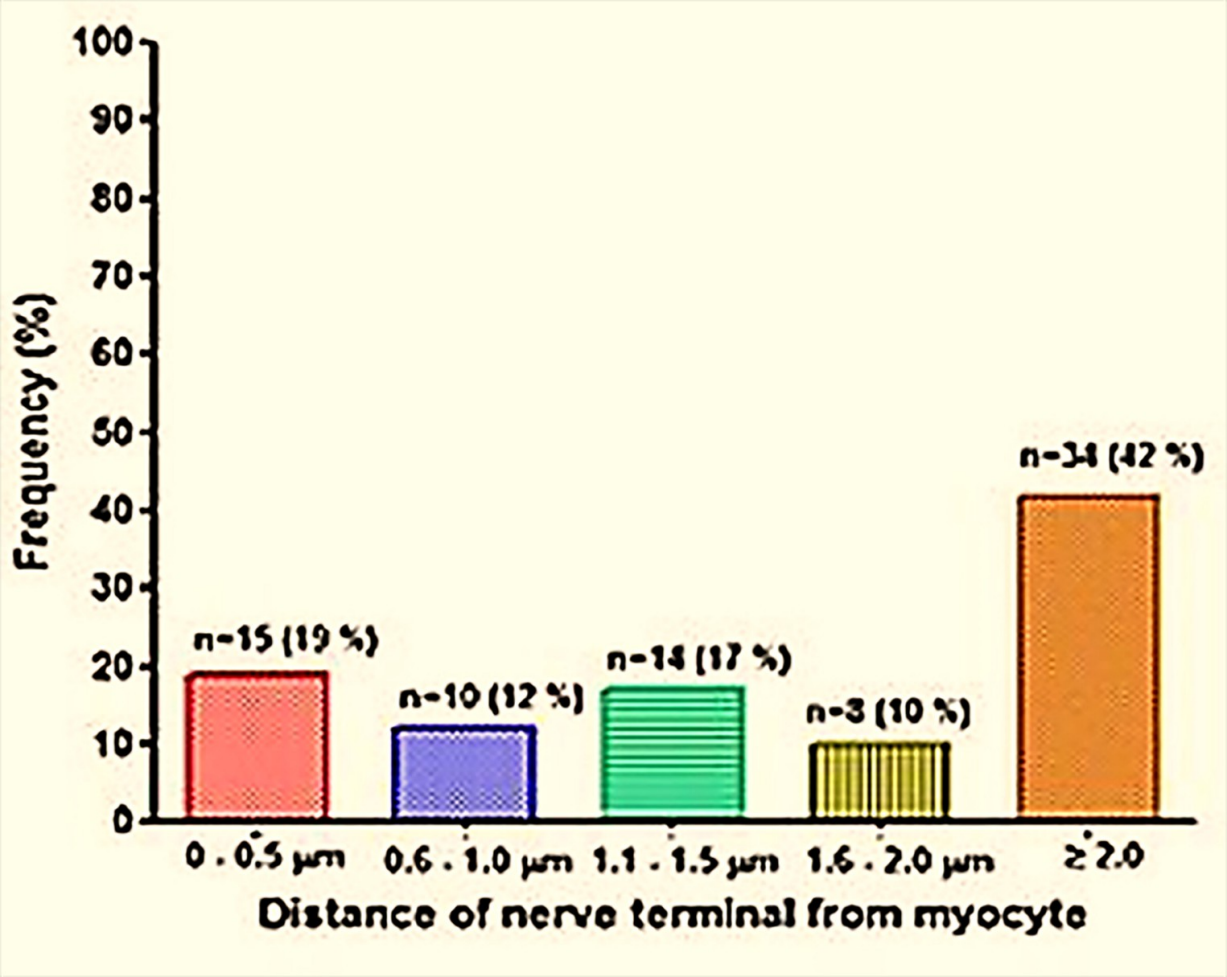
Histogram showing percentage representation of the number of nerve terminals with respect to their distance from the nearest myocyte.

TEM of mice LA revealed the presence of varicosities or nerve terminals confirming the earlier observed findings of immunohistochemistry. In total, all 81 nerve fibers observed were unmyelinated, had a Schwann cell present or not observed, and were predominately found within the media and adventitial layers. A higher number of these terminals (n = 47, 58%) were ≤ 2 μm away from the nearest myocyte, while 34 (42%) were ≥ 2.1 μm away from the nearest myocyte (Figs 5–8).

## Discussion and conclusion

Regulation of blood vessel and ultimately blood flow is under extensive control by the nervous system. Innervation of the fetal ductus arteriosus has been described early [10, 11]. On a macroscopic level, such fibers reach the ductus via the vagus nerve and branches originating from the thoracic sympathetic chain [6]. The present study demonstrates an extensive innervation of the LA in animals (mice and pigs) and in senescent humans. These findings were validated by two independent methods, i.e., immunolabeling with an antibody directed against a general neuronal marker protein (PGP9.5), and TEM. Immunofluorescence validated that the LA receives innervation by the intense immunoreactivity when the structure was subjected to antibody against PGP9.5. Protein gene product is a general neuronal marker, able to report the presence or absence of innervation within organs. TEM of mice LA revealed that such preterminal fibers are grouped in bundles and separated from the surrounding tissue by, at minimum, a perineurial sheath. Clearly, such nerve fiber bundles were also seen in the outer adventitia of the LA and in the nearby surrounding, but they will not be considered further here. TEM further revealed that about 30% of all terminals were less than 1 μm away from smooth muscle cells in the LA, which clearly differs from elastic arteries, where the distance between nerve terminals and smooth muscle cells is rarely less than 1 μm [12, 13]. Thus, neuromuscular units in the LA do neither fully match those of small arterioles, nor those of elastic arteries, but rather like muscular arteries. It is important to mention that a recent re-investigation into the morphology of the LA reported the presence of contractile muscular elements present within this structure [2]. The nerve fibers were found in proximity to the smooth muscle cells within the tunica media layer of this structure. The results of these experiments suggested that the LA receives dense innervation from nerve terminals rather than just representing a guide rail for nerve fibers passing by to reach the heart. Furthermore, this provides the first reliable study of innervation of the LA and makes room for further investigation into the neurochemistry of this innervation. This is crucial as the presence of nerve terminals may play a role in vessel compliance or impedance of the two great vessels related to this structure. The substances released by these fibers may also have an influence on cells and tissues in the immediate microenvironment of this structure.

### Future directions and clinical implications

- Does LA innervation influence or affect contractility of the muscular elements found within the structure?
- What is the make-up of the neurochemistry of LA innervation reported by this study?
- Could the dense innervation influence impedance/compliance of the great vessels to which it is attached? Pulmonary trunk impedance/ compliance has been reported to be the best prognostic parameter for patient survival in pulmonary hypertension.
